# Gel-Phase Microextraction Using Microfluidic-Directed Ultrashort Peptide Assemblies for the Determination of Drugs in Oral Fluids

**DOI:** 10.3390/ijms26209982

**Published:** 2025-10-14

**Authors:** M. Laura Soriano, Ana M. Garcia, Juan A. Garcia-Romero, Pilar Prieto, Aldrik H. Velders, M. Victoria Gomez

**Affiliations:** 1 Facultad de Ciencias y Tecnologías Químicas, Instituto Regional de Investigación Científica Aplicada (IRICA), Universidad de Castilla-La Mancha, Avda Camilo José Cela s/n, 13071 Ciudad Real, Spain; anam.garcia@uclm.es (A.M.G.); juana.garcia@uclm.es (J.A.G.-R.); mariapilar.prieto@uclm.es (P.P.); aldrik.velders@wur.nl (A.H.V.); 2 Affordable and Sustainable Sample Preparation (AS_2_P) Research Group, Departamento de Química Analítica, Instituto Químico para la Energía y el Medioambiente (IQUEMA), Universidad de Córdoba, Campus de Rabanales, Edificio Marie Curie, 14071 Córdoba, Spain; 3 Laboratory of BioNanoTechnology, Wageningen University, 6700 EK Wageningen, The Netherlands

**Keywords:** tripeptide hydrogels, microfluidics, drug extraction, oral fluids, peptide fibers, ^19^F NMR spectroscopy

## Abstract

This study introduces an innovative microfluidic-based approach for extracting drugs from oral fluids using self-assembled tripeptide hydrogels as sorbents. Peptide microfiber derived from the heterochiral tripeptide ^D^Leu-^L^Phe-^L^Phe was formed in situ within the 14 mm-long microchannel of a two-inlet microfluidic device. The methodology enables the laminar flow-driven mixing of buffer solutions, inducing hydrogel formation at their interface. The resulting fiber exhibited a well-defined morphology and β-sheet structure, confirmed by Raman spectroscopy and Thioflavin T fluorescence. The peptide fibers co-assembled successfully with 5-fluorouracil (5-FU) and naproxen (39.8 ± 1.4 nmol of 5-FU and 27.4 ± 6.6 nmol of naproxen per 112 nmol of peptide used to prepare the fiber), resulting in a molar ratio drug/peptide ratio of approximately 1:3 and 1:4, respectively, demonstrating versatility in drug entrapment. The use of the gel fiber as a sorbent phase was first assessed in buffer, and subsequently, the optimized method was applied to saliva. Adsorption studies under stopped-flow conditions showed a significant drug adsorption capability from buffered solutions by the pre-formed hydrogel (32.8 ± 0.9% of 5-FU and 36.4 ± 3.3% of naproxen per fiber preformed with 112 nmol of peptide), demonstrating their suitability as sorbent material. The extension of the methodology to simulated saliva samples allowed extraction of 36% of 5-FU by the fiber, as determined by ^19^F NMR spectroscopy on microcoils, which enabled us to work with the small volume of fluid extracted from the microfluidic device and provided clean spectra and quantitative results. These findings highlight the potential of this tripeptide hydrogel as a sorbent material for therapeutic drug monitoring and toxicological analysis via a simple, non-invasive and rapid approach for drug detection in oral fluids.

## 1. Introduction

The growing prevalence of pharmaceutical overdoses and the critical need for personalized cancer therapies have intensified demand for non-invasive drug monitoring platforms. Saliva analysis has emerged as a promising alternative to plasma testing due to its painless collection, reduced infection risks, and strong correlation with blood analyte levels [1,2].

While conventional sorbents like activated carbon show limitations in biocompatibility, enormous efforts have been made for simple designs of suitable lightweight materials enriched with hydrophilic and specific functional groups to target analytes, with high effectiveness towards drugs [3]. 3D networks like certain hydrogels offer several advantages such as sensors [4] or sorbents [5], including reversibility, ease of formation, flexibility and versatility and, in some cases, also drug adsorption capability by virtue of their tunable porous structure, programmable surface groups and modulated shape (on curved or flat solid supports or liquid media). The use of gels derived from natural materials for solid-phase microextraction has gained attention as a “green sample preparation” approach. This innovation is not limited to the development of hydrogel-based sorbents, but also extends to the design of new devices and extraction setups [6]. In this context, few examples are described, such as agarose-based gel-phase microextraction that has demostrated its usefulness for the detection of diclofenac and lactic acid on solid surfaces [7]. Moreover, alginate-based hydrogels were prepared using a novel lab-made device and utilized for gel-phase microextraction of fluoxetine and norfluoxetine in biological matrices [8].

The design of peptides capable of self-assembling into complex nanostructures has significantly expanded the scope for developing functional, responsive materials with tunable properties [9]. Supramolecular hydrogels formed through the self-assembly of ultra-short peptides have recently gained attention in bioanalytical applications, particularly as biosensors or drug release systems, owing to their intrinsic versatility and functional adaptability [9]. Although these materials have demonstrated molecular recognition capabilities, their potential as sorbents for extraction applications remains largely unexplored. Ultra-short peptides containing diphenylalanine (Phe-Phe) motifs are especially promising in this context [10], as they can interact with small molecules through both hydrogen bonding and π–π stacking interactions. These peptides can self-assemble into a wide range of nanostructures—including microtubules, nanofibers, nanospheres, and nanoribbons—driven by subtle molecular variations [11,12]. In this sense, the incorporation of D-amino acids into L-tripeptides has been shown to promote hydrogelation, leading to rapid gel formation under physiological conditions—an effect not observed in the corresponding all-L sequences [13]. This strategy, first reported in 2012 [14], has opened new avenues for the development of peptide-based hydrogels with enhanced performance in biomedical and analytical contexts. Notably, these hydrogels exhibit reversible pH-dependent gelation, forming robust gels at neutral pH and dissolving upon exposure to alkaline conditions (such as pH 12).

In this context, the heterochiral tripeptide ^D^Leu-^L^Phe-^L^Phe has demonstrated hydrogel formation under physiological conditions while maintaining biocompatibility. It possesses significant potential in controlled drug delivery systems due to its programmable self-assembly and molecular interaction capabilities. Several studies reveal its ability to form β-sheet-rich hydrogels that encapsulate chemotherapeutic agents like 5-fluorouracil (5-FU), enabling sustained release profiles through diffusion-mediated mechanisms [15]. This behavior aligns with broader trends in peptide-based drug delivery, where tripeptide nanostructures exhibit tunable release kinetics depending on their assembly pathways. The incorporation of aromatic residues facilitates π-stacking interactions with hydrophobic drugs such as naproxen, enhancing loading capacities while maintaining biocompatibility [16]. Though primarily investigated for therapeutic delivery, the peptide’s high surface-area nanostructures suggest latent potential as bio-sorbents, though this application remains unexplored compared to its established role in controlled release platforms.

A critical advancement in the application of this tripeptide is achieved by transitioning from conventional bulk gelation to microfluidic-directed assembly. Traditional vial-based gelation typically results in randomly oriented, heterogeneous networks that can limit the efficiency and reproducibility of the resulting material. In contrast, microfluidic systems provide unique advantages for triggering and controlling the self-assembly of supramolecular structures under continuous flow conditions [17]. The laminar flow regime and diffusion-dominated mixing at the interface of microchannels enable precise spatial and temporal control over peptide nucleation and fiber growth. As demonstrated in our previous work [17], the confinement within microfluidic channels facilitates the formation of highly elongated fibers with a high length-to-diameter ratio, which is not achievable under bulk conditions. This structural anisotropy, driven by pH-triggered gelation at the solution interface and shear alignment along the channel, leads to reproducible and continuous hydrogel architectures. At this point, we hypothesized whether such elongated fiber structures were particularly advantageous for sorbent applications, as they maximize the surface-area-to-volume ratio and therefore ensure prolonged contact between the hydrogel and the biological fluid -such as saliva- flowing through the microchannel. This not only enhances the efficiency of drug extraction but also ensures consistent performance due to the high reproducibility of the microfluidic fabrication process [17,18,19]. We think that by integrating heterochiral tripeptide hydrogels into microfluidic devices, would allow to engineer advanced sorbent materials with tailored morphology and functionality towards drugs.

In this work, we present for the first time the use of assembled ^D^Leu-^L^Phe-^L^Phe hydrogels formed by microfluidics as sorbent materials for the extraction of drugs from saliva. Some of us have previously explored the drug-release [15,16] or the optical [17] properties of such peptide-based hydrogels, in bulk and in microfluidic devices, respectively. However, the approach herein reported explores the advantages of microfluidic fabrication of the gel as sorbent -namely, the reproducible formation of highly elongated peptide fibers within a controlled microchannel environment- to maximize analyte contact and extraction efficiency. By integrating these supramolecular assemblies into cost-effective, 3D-printed microfluidic devices, we demonstrate their ability to selectively adsorb and extract model drugs, such as 5-FU, directly from complex biological matrices. Microcoil NMR was used to demonstrate the selective and sensitive 5-FU detection in saliva in an off-line analysis. This proof-of-concept establishes a new application for heterochiral tripeptide hydrogels as gel-phase media for microextraction, opening new avenues for non-invasive, efficient, and miniaturized drug monitoring in oral fluids.

## 2. Results and Discussion

### 2.1. Materials Choice and Objective

The tripeptide ^D^Leu-^L^Phe-^L^Phe has demonstrated the ability to co-assemble with various small molecules, making it a promising candidate for applications such as controlled drug release processes [15,16]. Nevertheless, its potential as a sorbent material for the removal or preconcentration of target molecules from complex matrices has not yet been explored. This addresses this gap by demonstrating the use of ^D^Leu-^L^Phe-^L^Phe as an adsorbent within a microfluidic device to capture drugs from biological samples.

We chose a microfluidic approach because it allows kinetic control of peptide fiber formation, a key advantage over conventional bulk hydrogel methods. This technology, previously developed by our group [17], enables the reproducible formation of elongated fibers with controlled morphology (see Figure S1), which is essential for maximizing the available surface area for adsorption.

As a proof of concept, we focused on two model drugs: 5-FU and naproxen. Saliva was selected as the biological matrix given the demand for non-invasive analytical methods and its ease of collection. Furthermore, microcoil-based ^19^F-NMR spectroscopy was used to detect 5-FU in small sample volumes collected from the microfluidic device and to reduce matrix interference that hinders the detection of 5-FU by other optical spectroscopic methods.

### 2.2. Materials (Microfluidic Device) Preparation and Fiber Characterization

**Figure 1:**
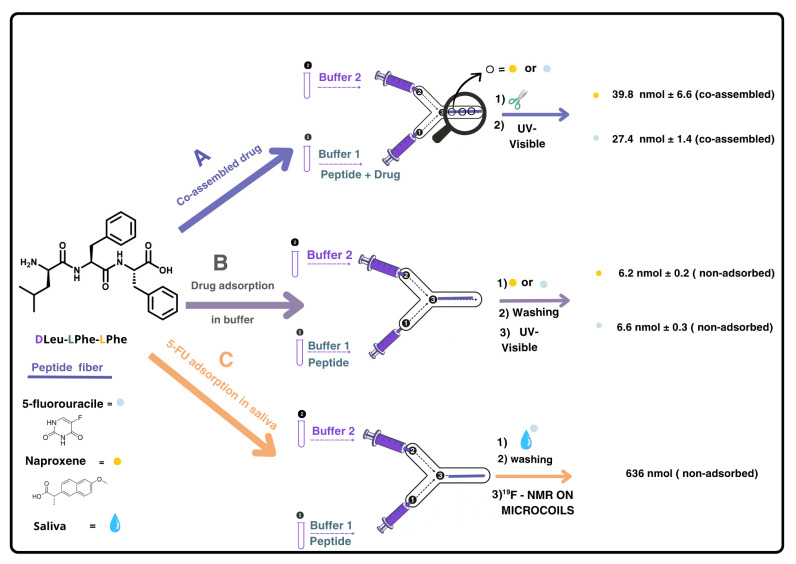
Scheme representing the experimental procedure carried out for: A: co-assembly of ^D^Leu-^L^Phe-^L^Phe with drugs (5-fluorouracile (5-FU) or naproxene), B: proof of concept use of the ^D^Leu-^L^Phe-^L^Phe supramolecular hydrogel as sorbent when the drugs were in buffer, and C: use of the hydrogel as a sorbent when the drug was in saliva. In all cases, 112 nmol of tripeptide was used to prepare the hydrogel fiber. In A, drugs were quantified after disassembling the peptide fiber whilst in B and C, drugs were quantified indirectly by measuring the washing solutions containing non-adsorbed drug. In B and C, the drugs (10 nmol and 1000 nmol, respectively) were loaded into the device once the hydrogel fiber was formed. In C, UV-visible spectroscopy could not be used as detection method due to saliva matrix interference; instead, ^19^F NMR was employed. The hydrogel fibers were formed in situ and remained inside the microchannel for the adsorption assays (B and C); they were not removed prior to injecting the drug-containing solultions.

PDMS/glass microfluidic devices were fabricated using our previously optimized replica molding protocol [17,18,19]. This two-inlet Y-chip enables controlled interfacial mixing of an alkaline peptide solution and a phosphate buffer containing the target drug, facilitating in situ hydrogel formation at an initial tripeptide concentration of 8 mM at the interface along the 1.4 cm-length central channel. This strategy enables precise control over the gel formation process, in contrast to bulk conditions [15,16] (Figure 1 and Figure S1).

Fluorescent microscopy confirmed that β-sheet formation is maintained during hydrogel self-assembly within the microdevice, consistent with prior reports [17], and the incorporation of dyes (e.g., thioflavin T (ThT) and rhodamine B (RhB)) did not alter this molecular arrangement. The formation of β-sheets during hydrogel self-assembly into the microdevice was also confirmed by Raman microspectroscopy, consistent with previous reports [17]. In addition, SEM analysis showed controlled one-dimensional alignment of fibrils, which is attributed to the microfluidic channel dimensions (Figure S1C). Thus, an optimized flow rate of 0.50 μL/min per inlet (total flow of 1.0 μL/min) was selected for the formation of twisted fibers approximately 250 μm in thickness (composed of bundled thinner fibrils) [17].

#### Co-Assembly of Peptide Fibers with Small Molecules Under Microfluidic Conditions

Once the microfluidic method enabled the reproducible formation of peptide fibers, preliminary studies were focused on assessing fiber assembly in the presence of drug molecules. The co-assembly of peptide fibers with small molecules was investigated under both bulk and microfluidic conditions. The tripeptide ^D^Leu-^L^Phe-^L^Phe has previously been shown to form hydrogels in the presence of structurally diverse drugs such as the hydrophilic 5-FU and the more hydrophobic naproxen [15,16]. In both cases, drug entrapment is primarily driven by H-bonding and π-π stacking interactions.

In the microfluidic setup, each drug was introduced via the phosphate buffer at alkaline pH, enabling co-assembled hydrogel fiber formation at the channel junction (Figure 1A). Drug incorporation was confirmed by disassembling the fibers under alkaline conditions and quantifying the released drug by UV-visible spectroscopy. After washing with pH ~6 buffer to remove unadsorbed drug, fibers were disassembled by injecting buffer at pH ~12, and the released 5-FU and naproxen were quantified (39.8 ± 1.4 nmol and 27.4 ± 6.6 nmol, respectively, per 112 nmol of tripeptide used to prepare each hydrogel, resulting in a drug:peptide molar ratio of 1:3 and 1:4, respectively). These experiments were performed in triplicate (see Section S1, ESI†, Tables S1 and S2). These results demonstrate that the microfluidic approach allows the co-assembly of the tripeptide fibers with both 5-FU and naproxen, while preserving the one-dimensional fibrillar structure reported in the absence of these drugs [17].

Notably, the microfluidic system enables the controlled formation of a single, continuous, elongated fiber directly within the channel and along its entire length (Figure S1). This level of spatial and morphological control is not achievable under bulk conditions, which typically lead to the formation of multiple, disordered fibers with heterogeneous surface areas [17]. Consequently, bulk were not pursued, because direct comparison between bulk and microfluidic approaches is neither feasible nor scientifically meaningful.

### 2.3. Drug Extraction and Analysis

We evaluated the use of ^D^Leu-^L^Phe-^L^Phe peptide fibers produced in microfluidic devices as a promising sorbent material for drugs extraction from saliva. This biological fluid is a non-invasive matrix increasingly employed in therapeutic drug monitoring because of its ease of collection and its ability to reflect unbound drug levels [20]. Saliva is particularly valuable for monitoring drugs such as 5-FU and naproxen, which occur at lower concentrations than in plasma but still provide clinically relevant information [20]. Microfluidic platforms are well suited for these applications, as they enable the reproducible production of fiber sorbents, facilitate efficient extraction of target analytes from small sample volumes, reduce reagent consumption, and allow seamless integration with downstream analytical techniques [21]. Previous studies have demonstrated the potential of microfluidic systems for detecting drugs of abuse in saliva, supporting their use in the present study for the determination of drugs in biofluids [22].

In our experimental setup, we evaluated the adsorption capacity of ^D^Leu-^L^Phe-^L^Phe peptide fibers for 5-FU and naproxen using both buffer and saliva matrices. The peptide fibers were first formed in the microchannel, as previously described, and then were exposed to standard drug solutions in buffer (10 nmol drug in phosphate-buffered solution at pH ~6) under stopped-flow conditions for 4 h. Preliminary on-flow experiments resulted in negligible drug retention. The amount of non-adsorbed drug was quantified using UV-visible absorption in both cases, yielding adsorption percentages of 32.87 ± 0.98% for 5-FU and 36.40 ± 3.28% for naproxen (see Section S2, ESI†, Tables S3 and S4) (Figure 1B). Having confirmed the adsorption of both drugs onto the ^D^Leu-^L^Phe-^L^Phe hydrogel, we extended our investigation to a biological matrix, using treated saliva samples spiked with known concentrations of 5-FU (Figure 1C). After repeating the experiments under the same setup and conditions used for the buffer, we encountered quantification challenges due to interfering proteins that absorb within the same spectral range as the selected analytes, which are also present in saliva samples (Figures S2 and S3).

**Figure 2:**
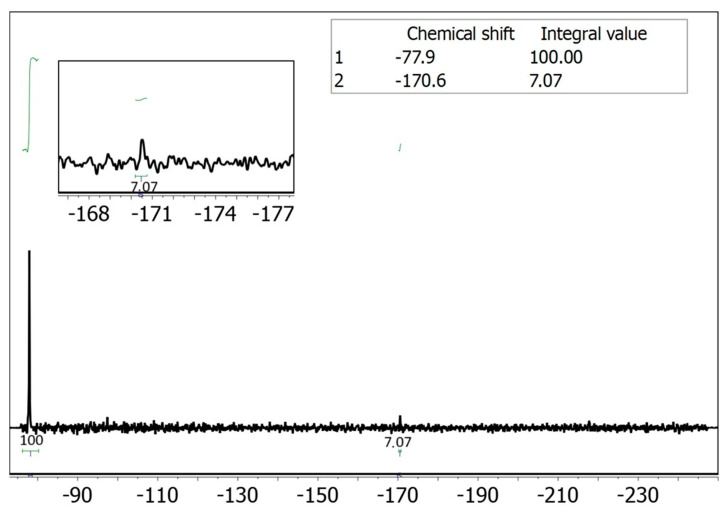
^19^F NMR spectrum of the non-adsorbed 5-FU in the presence of 215 mM of trifluoroethanol (TFE) in continuous flow regime at 0.7 μL/min [26,30]. The acquisition parameters are number of scans 1200, preacquisition delay 0.5 s and acquisition time 0.5 s, experiment time 20 min. The amount of TFE detected in the spectrum corresponds to 3 μmol if we consider the TFE concentration of 215 mM and a volume of 14 μL analyzed on continuous flow. Therefore, according to the integral value of 7.07, the amount of non-adsorbed 5-FU corresponds to 0.639 μmol. It corresponded to 361 nmol (36%) of 5-FU adsorbed considering that 1 μmol of compound was injected in the saliva matrix. The concentration of 5-FU at the ^19^F NMR spectrum corresponds to 46 mM.

Since UV-vis spectroscopy is hindered by interference from proteins present in saliva, we considered employing an alternative analytical technique such as NMR spectroscopy. In particular, fluorine-19 NMR (^19^F NMR) offers an attractive option, as it has well-established applications in drug detection [23,24], and, in this case, would provide clean spectra with a single signal corresponding to the drug. Moreover, performing a reaction in a microfluidic device inherently involves working with extremely small volumes of fluid. Therefore, to accurately monitor and analyze the outcome of such reactions, it is essential to use a detection system that is also compatible with micro-scale volumes. Conventional large-scale detection setups (i.e., a standard NMR tube) lack the sensitivity required for effective analysis. For this reason, reduced-diameter NMR detectors (namely microcoils) [25] have been used to detect the 5-FU present in saliva and adsorbed on the tripeptide. Our group has employed microcoils to overcome the inherent low sensitivity of NMR for small sample volumes in various applications [21,26,27,28,29]. Implementing this technique to quantify the adsorption efficiency of ^D^Leu-^L^Phe-^L^Phe peptide would enable selective quantification of the fluorine signal without interference from the biological matrix, eliminating the need for sample pre-treatment (Figure 1C). We carried out the adsorption experiments using saliva spiked with 5-FU by injecting 500 nmol (50 μL of a 10 mM solution of 5-FU in saliva) into the microfluidic device containing the peptide fiber. The following day, the 50 μL were eluted with 50 μL of buffer solution at pH ~6 to quantify the amount of 5-FU not adsorbed in the fiber (washing sample). A duplicate experiment was performed in parallel to obtain sufficient volume for the NMR analysis. The combined 100 μL eluted sample was introduced into the small-volume NMR setup [26,30], using a syringe pump after the addition of a known amount of trifluoroethanol (TFE) as an internal standard (see Section S3, ESI†). Integration of the fluorine signal in the spectrum corresponded to 639 nmol of non-adsorbed 5-FU, indicating that 36% of the injected 5-FU from saliva was adsorbed onto the peptide fiber (Figure 2).

These results illustrate that the adsorbing efficiency of the fiber was similar to the one obtained using a more diluted solution of 5-FU in buffer, being an indication of the promising analytical tool for the extraction and preconcentration of the drug. Increasing the length of the microchannel is expected to enhance the extraction or adsorption performance of the system, provided that the amount of peptide is sufficiently increased to allow the formation and elongation of peptide fibers along the extended channel. This theoretical expectation aligns with general principles of adsorption in microfluidic systems, where increased contact time and surface area typically promote higher adsorption efficiencies [31].

The concentration of analyte used is above to the one normally found in real biological samples (the typical concentration of 5-FU in saliva ranges from 0.1 to 28 µg/mL, depending on the patient [32]); therefore, this work serves as a proof of concept to use our systems for this purpose that could be applicable as a timely life-saving medical intervention to rapid identify/detect of overdose [33,34]. Once we have demonstrated the selectivity offered by ^19^F NMR spectroscopy on small-volume NMR techniques to quantify analytes in a complex, biological matrix, our current efforts are focused on extending this methodology to other samples where analytes are present at much lower concentrations.

## 3. Materials and Methods

### 3.1. Reagents and Instrumentation

All chemicals and biomolecules were used without further purification. Tripeptide ^D^Leu-^L^Phe-^L^Phe was purchased from DG peptides Co., Ltd., Hangzhou, China. Sodium hydroxide and hydrochloride acid were supplied by Scharlau (Barcelona, Spain) and Sigma Aldrich (St. Louis, MO, USA), respectively. Phosphate buffer solutions at pH 12 and 5.8 were supplied by Fisher Scientific (Waltham, MA, USA).

pH bench meter (electrochemical sensor XS) was purchased from VioLab (Covo, Italy).

High purity Milli-Q-water (MQ water) with a resistivity greater than 18 M·Ω·cm was obtained from an in-line Millipore RiOs/Origin system (Millipore RiOs/RiOs-Origin, Molsheim, France).

Saliva sample preparation: Saliva from a healthy person was collected and spiked with known concentrations of 5-FU. Standard solutions of both drugs as the controls were also prepared at the desired concentrations in adjusting phosphate buffer at the pH of saliva (i.e., pH ~6).

Scanning electron microscopy (SEM) images were obtained on a JEOL JSM 6335F microscope (JEOL, Akishima, Tokyo, Japan) working at 10 kV. A piece of PDMS device containing the corresponding fiber was deposited onto a glass substrate prior to SEM imaging. In the case of the hydrogel, the sample was transferred to a glass slide and dried under vacuum before analysis.

The NMR experiments on planar spiral microcoils were carried out in a 11.7 T narrow-bore Oxford instruments magnet (Oxford instruments, Abingdon, Oxfordshire, UK) (500 MHz 1H Larmor frequency and 470 MHz 19F Larmor frequency) INOVA NMR spectrometer (Agilent, Santa Clara, CA, USA). The microfluidic NMR chips used were described in previous publications from our group [26,28,30,35]. It consisted of a spiral planar microcoil located on top of a microfluidic glass substrate. Each planar spiral microcoil consists of 32 turns with a width and separation of 20 µm and an inner diameter of 250 µm, yielding a total detection volume of 25 nL. No tuning or matching capacitors were used in the RF circuit due to its broad-band property [26,30,36]. Fused-silica capillaries of 75 µm inner diameter were connected to the inlets of the microfluidic NMR chip to load the sample into the microchip and work on continuous flow regime. All experiments were acquired in non-locked mode, adjusting shims on the 1H solvent peak. Standard pulse sequences from the VnmrJ software were used for all NMR experiments (VnmrJ, Version 2.3 Revision A, Chempack 5.1 (2013)). Spectra were processed with MestReNova 14.2.2 software (Mestrelab Research, S. L., Santiago de Compostela, Spain) [26,37].

### 3.2. In-Flow Gelation of the Tripeptide in Microfluidic Devices

#### 3.2.1. Peptide Fiber Formation

The heterochiral tripeptide ^D^Leu-^L^Phe-^L^Phe was dissolved in sodium phosphate buffer 0.1 M at pH 11.8 (buffer 1) at 8 mM concentration. The same volume of sodium phosphate at 0.1 M, pH 5.8 was used (buffer 2). In a typical flow experiment, the two solutions A and B were carefully mixed with a flow rate of 1 µL/min controlled by a separate syringe pump, in order to fill the middle flow channel at a time. The flow rate of 1 µL/min was selected to reach an incubation period of 14 min in devices with an inner channel volume of 14 μL. The intersection of both solutions inside the channels was monitored and their mixture led to tripeptide fiber formation inside the device, thanks to a pH trigger [17].

#### 3.2.2. Peptide Fiber Formation in the Presence of Drugs (5-FU or Naproxen)

To achieve fiber formation in the presence of drugs, they were incorporated with the peptide in buffer 1 (inlet 1). In the case of 5-FU, ^D^Leu-^L^Phe-^L^Phe (8 mM) was dissolved in 250 μL of sodium phosphate buffer (0.1 M) at pH 12.0 (buffer 1) in the presence of 5-FU (final concentration of 13 mM). In the case of naproxen, it was previously dissolved in freshly prepared 1 M NaOH (5 min sonication) and added to buffer 1 where its final concentration was 26 mM. On the inlet 2, only a phosphate-buffered solution (pH 4.5 in the presence of drugs instead of a pH 5.8 as mentioned above) was used (buffer 2). Both solutions were filtered (0.2 μm) prior to use. Then, they were loaded in the corresponding syringe controlled by a pump and mixed inside the microfluidic device under the same conditions described for peptide fiber formation in the absence of drug.

### 3.3. Microfluidic Device Preparation

The procedure has been reported previously [17,18,19]. A 3D mold was designed in Tinkercad (Autodesk, Inc., San Francisco, CA, USA) and printed with an mSLA 3D printer (Prusa Research, Prague, Czech Republic) or an SLA one (Formlabs Form 3, Inc., Somerville, MA, USA) using Prusa tough resin and Formlab clear V4 resin, respectively. After 3D printing, the mold was washed with ethanol and isopropanol and then allowed to dry. For easy peeling of the PDMS, the 3D printed molds were covalently coated with trichloro (1H,1H,2H,2H-perfluorooctyl)silane (PFOTS, 97%) using chemical vapor deposition (CVD) in a vacuum desiccator (JP Selecta S. A, Barcelona, Spain). The 3D printed structure was air plasma-activated for 30 s, and then, it was placed in a desiccator with a vial of 100 µL of PFOTS and high vacuum was applied using the pump. For the plasma treatment, an Inseto Plasma Etch, Inc. (Carson City, NV, USA) PE-25 benchtop air plasma cleaner was used at its maximum RF plasma power of 100 W with an air flow of ~10 cc min^−1^, which allowed for a vacuum pressure of 200–250 mTorr within the chamber during plasma treatment. The desiccator was then left under a static vacuum for overnight CVD of PFOTS. After deposition, the 3D printed structure was removed from the desiccator and left in the oven at 70 °C for 1 h, then it was washed with ethanol and isopropanol.

PDMS replicas were prepared by pouring the PDMS degassed mixture (10:1 silicone elastomer to curing agent) on the 3D printed PFOTS coated mold. After overnight curing at 70 °C, the PDMS was demolded and sonicated in ethanol for 4 min to remove the low molecular weight and unreacted PDMS chains. The PDMS replica was then dried with nitrogen. An inlet and outlet were then created on the edges of the microchannel features by puncturing through the PDMS with a 1.0 mm ∅ or 1.5 mm ∅ punch (KAI, Tokyo, Japan).

The PDMS replica was placed facing upwards in the plasma oven with a clean microscope glass slide. The slide and the PDMS replica were treated in a plasma oven (see above for details). After this, the PDMS replica was firmly placed on the glass slide, and the microfluidic was left in the oven at 70 °C overnight.

### 3.4. Extraction Procedure and Analysis

All absorption and elution experiments and analyses of the retained drugs were performed in triplicate.

#### 3.4.1. Absorption Experiments

##### Adsorption Using Solutions of Drugs in Buffer

Peptide fiber was formed in the microfluidic devices as described. Then, 50 μL of either 5-FU or naproxen at 200 μM (10 nmol) in phosphate buffer at pH 6 were flowed through the two inlets at a flow rate of 1 mL/min controlled by a separate syringe pump (pH 6.1 ± 0.1 at which the fiber is stable). The drug solution was left in stopped flow for 4 h and then another 50 μL of solution were flowed to collect the 50 μL that were inside the device (see Section S2 ESI†, Tables S3 and S4). The samples collected were diluted to a final volume of 450 μL and analyzed by UV–visible spectroscopy to quantify the amount of 5-FU or naproxen, according to the calibration curves previously obtained at this pH (Figures S4–S7).

##### Adsorption Using Solutions of Drugs in Saliva and ^19^F NMR Detection

500 nmol (50 μL of 10 mM 5-FU in saliva) was injected into the microfluidic device containing the peptide fiber. The conditions for drug adsorption were the same as indicated above for adsorption of drugs in buffer solutions. After adsorption, 50 μL were eluted using a pH 6 buffer solution. A parallel experiment was conducted to obtain 100 μL total volume for the ^19^F NMR analysis; briefly, the 100 μL eluted sample was injected into a small-volume NMR setup [26,30]. Trifluoroethanol (TFE) was added as an internal standard (see Section S3, ESI†).

#### 3.4.2. Elution Experiments

Elution of the drug embedded in the peptide fiber was performed at a total flow speed at the middle channel of 10 μL/min, using phosphate buffer at pH 12 in order to disassemble the tripeptide fiber. Experiments were performed in triplicate. The values were averaged, and the standard deviation calculated with Excel. After each experiment, the devices were carefully washed with a solution of NaOH 0.5 M and then with distilled water (see Section S1, ESI†).

### 3.5. Calibration Curves from UV Analysis

Calibration curves for both drugs 5-FU and naproxen were constructed at pH 6 (to monitor the amount of drug after washing or adsorbed) and 12 (after elution of drug from the peptide fiber). For 5-FU, standard solutions were prepared in the concentration range of 10–250 µM. The absorbance of these solutions was measured at 264 nm using a UV-visible spectrophotometer (Jasco, V-750, Tokyo, Japan) (Figures S4 and S5, ESI†). Similarly, for naproxen, standard solutions were prepared in the concentration range of 10–250 µM and their absorbance was measured at 330.5 nm (Figures S6 and S7, ESI†).

Linear regression analysis was performed to establish the relationship between absorbance and concentration for both drugs at each pH value. The resulting calibration curves were used to determine the concentrations of 5-FU and naproxen in subsequent experiments.

## 4. Conclusions

In this work, we demonstrate the applicability of ^D^Leu-^L^Phe-^L^Phe peptide fibers for drug extraction from oral fluids. The controlled assembly of these fibers within microchannels allowed selective adsorption of drugs under simulated saliva conditions, highlighting their potential for drug detection in complex biological matrices. Notably, stopped-flow conditions within microfluidic devices were essential to achieve measurable drug retention, whereas continuous flow experiments showed negligible adsorption. On the other hand, conducting this type of study under bulk conditions requires a much larger amount of tripeptide and is more challenging due to the increased difficulty in handling and controlling the system.

The peptide fibers exhibited significant adsorption capacities in buffer solutions, with rates of 32.9 ± 1.0% for 5-FU and 36.4 ± 3.3% for naproxen (per 112 nmol of peptide used for fiber formation). Extending this methodology to real saliva samples, we overcame analytical challenges by employing ^19^F-NMR spectroscopy on microcoils as a detection method, particularly for 5-FU detection. It enabled selective and sensitive quantification of 5-FU without sample pre-treatment, measuring an adsorption rate of 36%. Such a non-invasive approach is particularly valuable for monitoring fluoride-containing drugs like 5-FU in easily accessible biological fluids at low absolute amounts.

Overall, the integration of heterochiral tripeptide hydrogels into microfluidic devices enables the design of advanced sorbent materials with controlled morphology and tailored functionality, opening new possibilities for innovative bioanalytical and therapeutic applications. In particular, our research demonstrates the potential of ^D^Leu-^L^Phe-^L^Phe fiber in gel-phase microextraction for drug capture and monitoring in saliva matrices. This approach opens new avenues for the development of sensitive, selective, and non-invasive methods for therapeutic drug monitoring, with potential applications in personalized medicine and drug development. Further research is needed to fully evaluate the method’s efficacy in real-world clinical settings.

## Data Availability

The original contributions presented in this study are included in the article/Supplementary Material. Further inquiries can be directed to the corresponding authors.

## Supplementary Materials

The following supporting information can be downloaded at: https://www.mdpi.com/article/10.3390/ijms26209982/s1. References [17,30,38,39] are cited in the Supplementary Material.
