# Supplementary material for: Gel-Phase Microextraction Using Microfluidic-Directed Ultrashort Peptide Assemblies for the Determination of Drugs in Oral Fluids
Source: Int J Mol Sci. 2025 Oct 14;26(20):9982. doi: 10.3390/ijms26209982 (PMC12564166; doi:10.3390/ijms26209982)
Supplement: Supplementary file 1 [file ijms-26-09982-s001.zip › ijms-3806189-supplementary.pdf]

Supporting Information

Gel-Phase Microextraction Using Microfluidic-Directed Ultrashort Peptide Assemblies for the Determination of Drugs in Oral Fluids

M. Laura Soriano [1,2,*], Ana M. Garcia [1], Juan A. Garcia-Romero [1], Pilar Prieto [1], Aldrik H. Velders [1,3] and M. Victoria Gomez [1,*]

[1] Facultad de Ciencias y Tecnologías Químicas, Instituto Regional de Investigación Científica Aplicada (IRICA), Universidad de Castilla-La Mancha, Avda Camilo José Cela s/n, 13071 Ciudad Real, Spain; anam.garcia@uclm.es (A.M.G.); juana.garcia@uclm.es (J.A.G.-R.); mariapilar.prieto@uclm.es (P.P.); aldrik.velders@wur.nl (A.H.V.)

[2] Affordable and Sustainable Sample Preparation (AS_2P) Research Group, Departamento de Química Analítica, Instituto Químico para la Energía y el Medioambiente (IQUEMA), Universidad de Córdoba, Campus de Rabanales, Edificio Marie Curie, 14071 Córdoba, Spain

[3] Laboratory of BioNanoTechnology, Wageningen University, 6700 EK Wageningen, The Netherlands

* Correspondence: laura.soriano@uco.es (M.L.S.); mariavictoria.gomez@uclm.es (M.V.G.)

S1. Elution experiments

Elution of the drug embedded in the peptide fiber was performed at a total flow speed at the middle channel of 10 µL/min, using phosphate buffer at pH 12 in order to disassemble the tripeptide fiber. Experiments were done in triplicate. The nmol released was calculated using calibration curves performed for each drug at different concentrations (**Fig. S4** and **Fig. S6**). The values were averaged, and the standard deviations were calculated using Excel (see **Tables S1 and S2**). After each experiment, the devices were carefully washed with a solution of NaOH 0.5 M and then with distilled water.

Table S1. Quantification of 5-fluorouracile (5-FU) co-assembled in the tripeptide fiber (per 112 nmol of tripeptide).

**Experiment**	**Volume analyzed (µL)**	**Concentration (µM)**	**nmol released**
Exp1	500	79.194	39.6
Exp2	500	77.108	38.6
Exp3	500	82.615	41.3
			**Average = 39.8**<br>**STD = 1.4**

Table S2. Quantification of naproxen co-assembled in the tripeptide fiber (per 112 nmol of tripeptide).

**Experiment**	**Volume analyzed (µL)**	**Concentration (µM)**	**nmol released**
Exp1	2350	8.697	20.4
Exp2	430	65.545	28.2
Exp3	370	90.746	33.6
			**Average = 27.4**<br>**STD = 6.6**

S2. Adsorption experiments and quantification by UV-visible spectroscopy

a. Adsorption using solutions of drugs in buffer

To start, the peptide fiber was formed in the microchannels as described previously [17][+]. Then, the fiber was loaded with 10 nmol of drug (50 µL of a 200 µM solution of drug in buffer at pH ~6). The solution was injected into the microfluidic device and left in a stopped flow for 4 hours. Stopped-flow conditions were needed to allow adsorption as previous experiments carried out on-flow led to negligible drug retention (data not shown). After this time, those 50 µL were eluted and collected in a vial. Each experiment was performed in triplicate. The amount of non-adsorbed drug was quantified using UV-vis absorption in both cases, using calibration curves performed at different concentrations (**Figure S5** and **Figure S7**), leading to percentages of adsorption of 32.76 ± 0.97 % for 5-FU and 36.40 ± 3.28 % for naproxen, respectively (see **Tables S3 and S4**, respectively).

Table S3. Quantification of 5-FU adsorbed in the tripeptide fiber (per 112 nmol of tripeptide).

Experiment	$C_{solution\ injected}$ (µM)	nmol injected	nmol non-adsorbed*	% Adsorption
Exp1	191.241	9.562	6.411	32.9
Exp2	188.124	9.406	6.410	31.9
Exp3	210.990	10.500	6.983	33.5
			**Average = 6.6**<br>**STD = 0.3**	**Average = 32.8**<br>**STD = 0.9**

*from the UV quantification using the corresponding calibration curve.

Table S4. Quantification of naproxen adsorbed in the tripeptide fiber (per 112 nmol of tripeptide).

Experiment	$C_{solution\ injected}$ (µM)	nmol injected	nmol non-adsorbed*	% Adsorption
Exp1	196.421	9.821	6.018	38.7
Exp2	192.253	9.613	6.337	34.1
			**Average = 6.2**<br>**STD = 0.2**	**Average = 36.4**<br>**STD = 3.3**

*from the UV quantification using the corresponding calibration curve.

b. Adsorption using solutions of 5-FU in saliva

Saliva treatment and UV-visible analysis: several attempts were made to treat saliva in order to make it suitable for the quantification of adsorbed drug by UV-visible analysis, including dilution with Milli-Q water, centrifugation or protein digestion treatment (attempts 1-8) [38-39][2],[3]. However, any of them worked and led to noise in the UV-visible spectra. Therefore, we envisioned NMR spectroscopy as analytical technique to detect 5-FU in saliva samples as reported below.

Attempts 1-8:

Attempt 1 (**Figure S2**): Saliva was diluted with distilled H_2O (H_2O/saliva, 1/2). The UV spectrum shows a lot of scattering and some components that absorb in the region 200-300 nm.

Attempt 2 (**Figure S2**): 300 μL of sample from attempt 1 were diluted with 200 μL of distilled H_2O. Same problems in the spectrum than attempt 1.

Attempt 3 (**Figure S2**): Sample was prepared as in attempt 2 was intended to filter with a 0.45 μm filter, but it was impossible because the filter was completely blocked.

Attempt 4 (**Figure S2**): Saliva was centrifuged at 7000 rpm for 10 minutes. Then, it was diluted with distilled H_2O (H_2O/saliva, 1/2) and filtered with a 0.45 μm filter. The UV spectrum shows some components that absorb in the region 200-300 nm, but scattering disappears.

Attempt 5 (**Figure S2**): Sample was prepared as in attempt 4 but spiked with a 5-FU at a final concentration of 200 μM. The UV spectrum is similar to that for attempt 4, therefore we cannot use this analytical technique to quantify 5-FU in the saliva samples.

Attempt 6 (**Figure S3**): 4 mL of saliva were mixed with 800 μL of 1 M phosphoric acid and vigorously stirred to favor proteins precipitation. 250 μL of treated saliva were mixed with 250 μL of distilled H_2O. The UV spectrum shows interferences that absorb in the region 200-300 nm.

Attempt 7 (**Figure S3**): Take 2 mL of saliva with treatment done in attempt 6 and centrifuge at 7000 rpm for 20 minutes. The UV spectrum shows interferences that absorb in the region 200-300 nm (**Figure S3**).

Attempt 8 (**Figure S3**): Take 2 mL of saliva with treatment done in attempt 7 and filtered with a 0.45 μm filter. The UV spectrum is similar to the one obtained for attempt 7.

S3. NMR analysis to quantify adsorption by ^{19}F-NMR on microcoils.

A previous setup based on small-volume NMR with the use of microcoils developed by our group was employed [30][4]. The washing solution was the chosen one to be pumped to the microfluidic system for its analysis due to the higher volume compared to the elution solution. The volume used to wash the peptide fiber formed in a single microdevice was 50 µL, whist the volume used for the elution (to disassemble the fiber) was 30 µL. In addition, according to our control experiments, the amount of 5-FU present in the washing solution should be higher than in the elution solution, which facilitates its detection.

Trifluoroethanol (TFE) was used as an internal standard with a concentration of 215 mM. Two experiments were run in parallel to have double volume to facilitate the analysis in the NMR set up, as explained above. Then, 50 µL of saliva spiked with 5-FU, 10 mM, was loaded in each microfluidic device containing the peptide fiber (112 nmol of tripeptide). The day after, those 50 µL were eluted with a buffer solution of pH 6 to quantify the amount of 5-FU not adsorbed in the fiber defining the so-called washing solution. To the final 100 µL the corresponding amounts of TFE (215 mM) were added prior to be pumped to the NMR setup for quantification purposes.

Figures

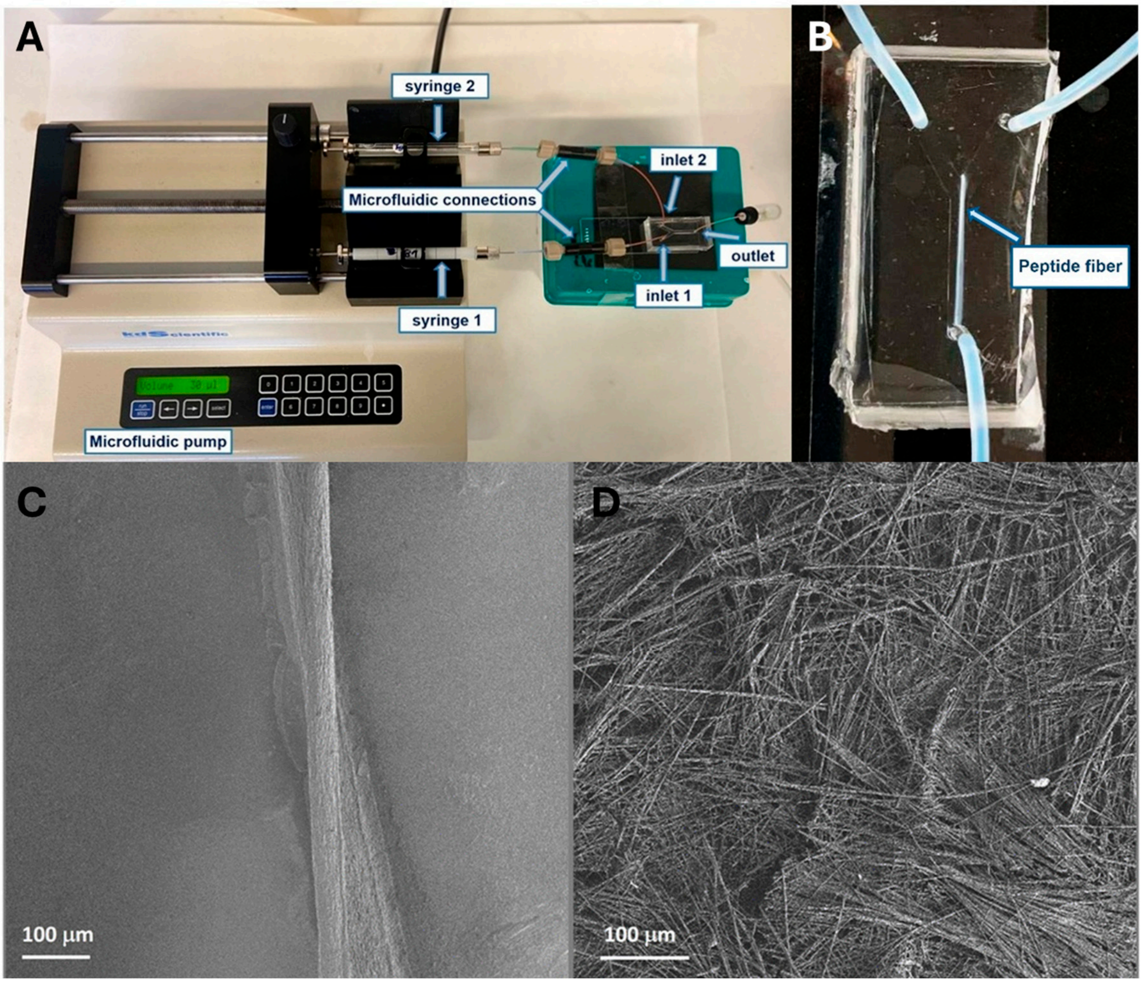

Figure S1. Microfluidic setup (**A**) to form the elongated fiber; an enlarged view of the Y-shaped device where the fiber visibly forms at the interface as the two solutions meet though the inlets (**B**); and their SEM micrograph when prepared in the microfluidic device (**C**) in comparison with the fiber formation under classical conditions (**D**).

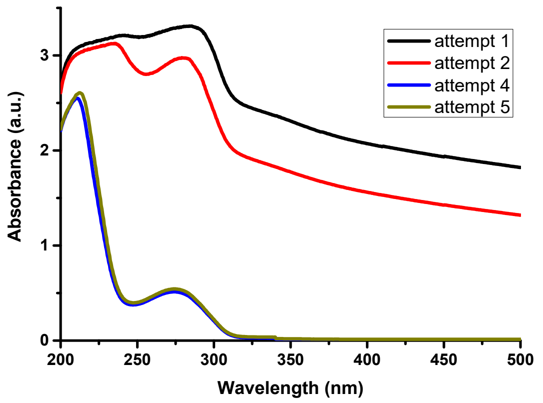

Figure S2. UV spectra for saliva samples described above (attempts 1-5).

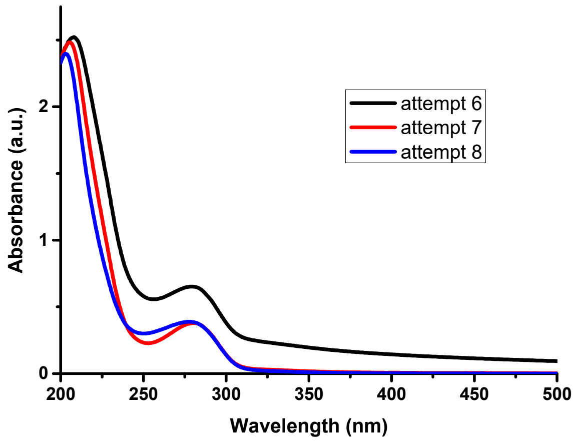

Figure S3. UV spectra for saliva samples described above (attempts 6-8).

.

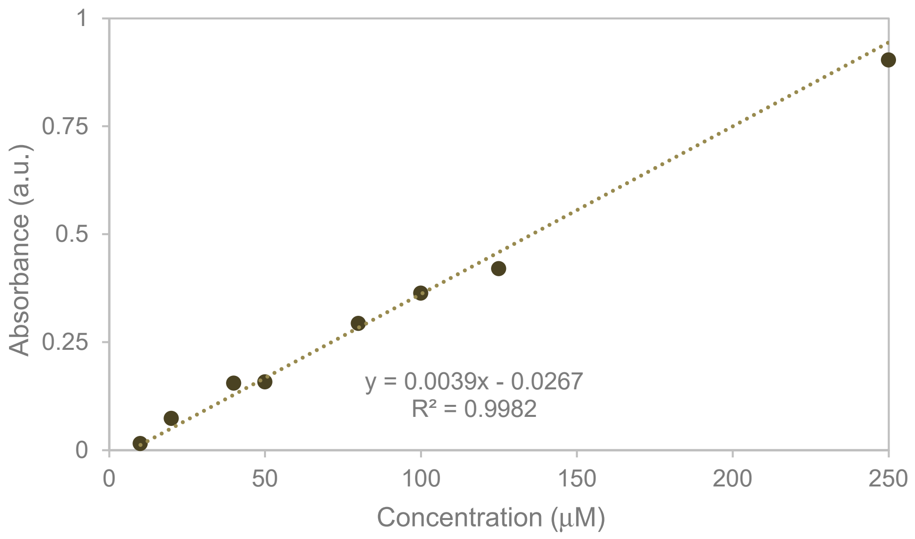

Figure S4. Calibration curve of 5-FU (10-250 µM) at pH 12 recording the absorbance intensity at 264 nm.

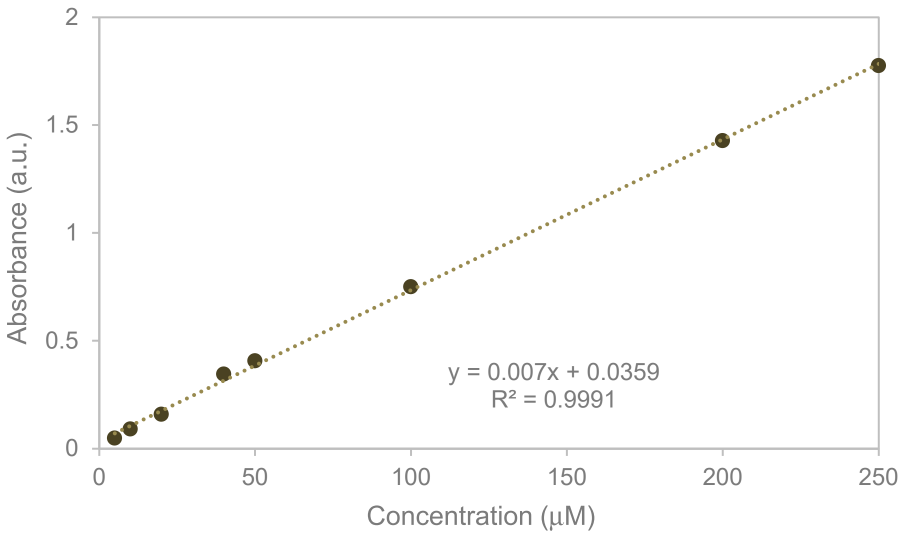

Figure S5. Calibration curve of 5-FU (10-250 µM) at pH 6 recording the absorbance intensity at 264 nm

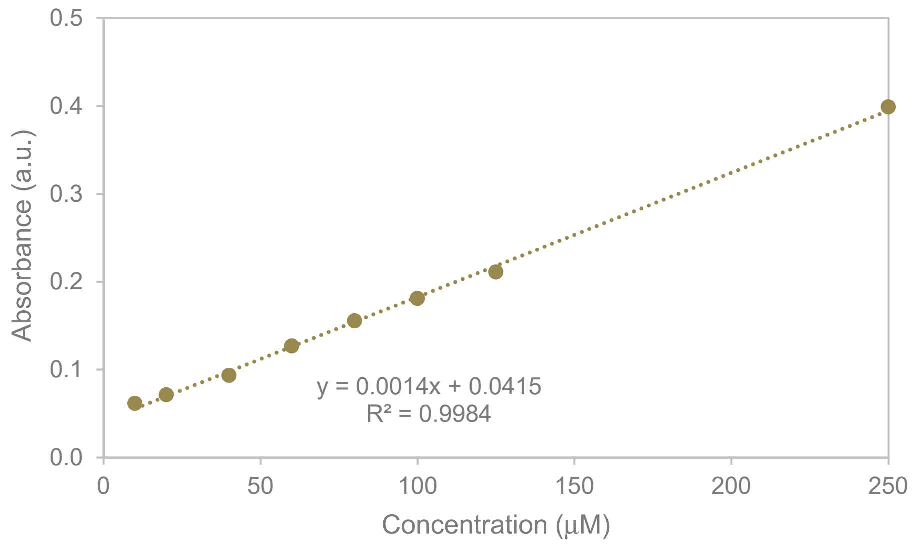

Figure S6. Calibration curve of naproxen (10-250 µM) at pH 12 recording the absorbance intensity at 330.5 nm

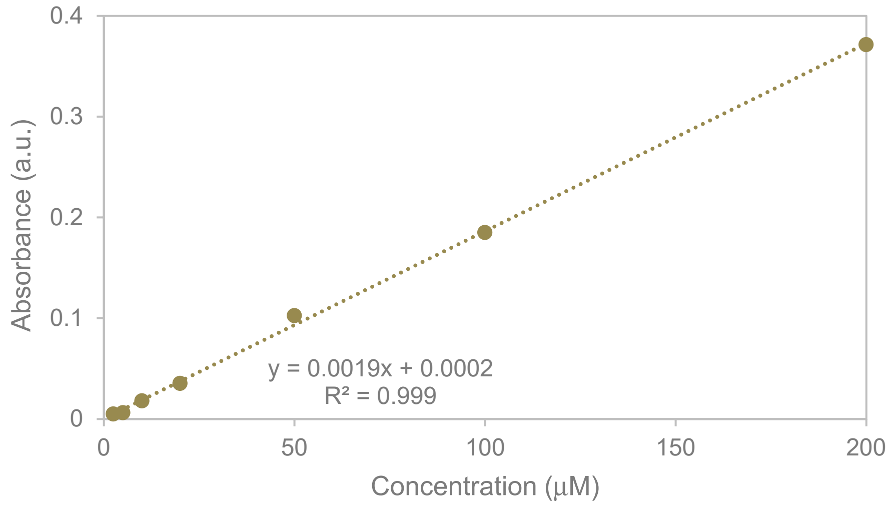

Figure S7. Calibration curve of naproxen (10-250 µM) at pH 6 recording the absorbance intensity at 330.5 nm

References

[17] Garcia, A.M.; Garcia-Romero, J.A.; Mejias, S.H.; Prieto, P.; Saggiomo, V.; Velders, A.H.; Soriano, M.L.; Ruiz-Díez, V.; Cabanillas-González, J.; Gomez, M.V. Microfluidic-Driven Short Peptide Hydrogels with Optical Waveguiding Properties. J. Mater. Chem. C 2024, 12, 6027–6034, doi:10.1039/d4tc00282b.

[30] Fratila, R.M.; Gomez, M.V.; Sýkora, S.; Velders, A.H. Multinuclear Nanoliter One-Dimensional and Two-Dimensional NMR Spectroscopy with a Single Non-Resonant Microcoil. Nat. Commun. 2014, 5, doi:10.1038/ncomms4025.

[38] Hines, E.P.; Calafat, A.M.; Silva, M.J.; Mendola, P.; Fenton, S.E. Concentrations of Phthalate Metabolites in Milk, Urine, Saliva, and Serum of Lactating North Carolina Women. Environ. Health Perspect. 2009, 117, 86–92, doi:10.1289/ehp.11610.

[39] Patsalos, Philip N.; Spencer, Edward P.; Berry, D.J. Therapeutic Drug Monitoring of Antiepileptic Drugs: A 2013 Update. Ther. Drug Monit. 2013, 35, 1–47, doi:10.1097/FTD.0b013e31827c11e7.
